# Facile Sulfurization under Ambient Condition with Na_2_S to Fabricate Nanostructured Copper Sulfide

**DOI:** 10.3390/nano11092317

**Published:** 2021-09-06

**Authors:** Eunseo Hwang, Yoonsu Park, Jongbae Kim, Taejong Paik, Don-Hyung Ha

**Affiliations:** School of Integrative Engineering, Chung-Ang University, Seoul 06974, Korea; nseo4236@cau.ac.kr (E.H.); ung2150@cau.ac.kr (Y.P.); jong3070@cau.ac.kr (J.K.); paiktae@cau.ac.kr (T.P.)

**Keywords:** sulfurization, Na_2_S, copper sulfide, nanostructure, Cu film

## Abstract

The sulfurization reaction was investigated as a promising fabrication method for preparing metal sulfide nanomaterials. Traditional sulfurization processes generally require high vacuum systems, high reaction temperatures, and toxic chemicals, utilizing complicated procedures with poor composition and morphology controllability. Herein, a facile method is reported for synthesizing nanostructured copper sulfide using a sulfurization reaction with Na_2_S at room temperature under non-vacuum conditions. Moreover, we demonstrate that the morphology, composition, and optical properties of nanostructured copper sulfides could be controlled by the Na_2_S solution concentration and the reaction time. Nanostructured copper sulfides were synthesized in nanospheres, nanoplates, and nanoplate-based complex morphologies with various oxidation states. Furthermore, by comparing the optical properties of nanostructured copper sulfides with different oxidation states, we determined that reflectivity in the near infrared (NIR) region decreases with increasing oxidation states. These results reveal that the Na_2_S solution concentration and reaction time are key factors for designing nanostructured copper sulfides, providing new insights for synthesis methods of metal sulfide nanomaterials.

## 1. Introduction

Metal sulfide nanomaterials have attracted significant attention due to their unique optical properties and their numerous applications in photocatalysis [1], energy conversion [2], optoelectronic devices [3], biomedicine [4], and light-emitting devices [5]. The synthesis methods play a vital role in controlling the properties of metal sulfides for maximizing their performance in applications. Numerous studies have reported various synthesis methods for metal sulfide nanomaterials, including thermal evaporation [6], electro-beam evaporation [7], spray pyrolysis [8], chemical bath deposition [9], electrochemical deposition [10], hydrothermal methods [11], solvothermal methods [12], sono-chemical methods [13], and sulfurization [14,15]. Among them, sulfurization is one of the most effective methods for preparing metal sulfide nanoparticles with well-controlled compositions, and can improve the surface properties, such as electronic properties, hydrophobicity, and corrosion resistance [14]. Such sulfurization refers to the modification or transformation of metal-based materials due to exposure to sulfur compounds with various oxidation states and generally utilizes metals with strong affinities for sulfide [16]. Although sulfurization has numerous advantages, further development and investigation should be conducted due to the limitations of the current techniques. For example, the evaporation methods for sulfurization require a vacuum chamber and a carrier gas to prevent impurities and require high reaction temperatures (150–500 °C) [17,18,19]. Another example is the aqueous-solid phase sulfurization process (solution process), which involves reactions between dissolved sulfur species and solid metal materials. Generally, the solution process employs toxic chemicals as sources of sulfur, such as hydrogen sulfide [20] and ammonium sulfide [21], which are extremely volatile, hindering the precise control of the reaction.

To overcome the limitations of sulfurization reactions employing traditional methods, the solution process with Na_2_S might be a suitable alternative technique. Sodium sulfide is one of the most widely used sulfur sources in various solution processes because of its non-toxic nature, high mobility of sodium ions, earth abundance, and low cost [14,22,23]. Various syntheses with Na_2_S have been reported for nanostructured metal sulfides, demonstrating size tuning through the control of the reaction temperature and time [24]. However, most of those reactions require high reaction temperatures and show poor controllability of morphology and composition for metal sulfides. Thus, simple synthesis methods capable of controlling the metal sulfides’ morphology and composition should be developed through rational design.

Particularly, due to their stoichiometry-dependent band gap, copper sulfide nanomaterials have shown great potential as advanced p-type semiconductors in various applications with unique optical properties [25,26]. By controlling the compositions, sizes, crystalline phases, and morphologies of nanostructured copper sulfides, excellent performance [27] has been achieved in photocatalysis [28], energy conversion [29], optoelectronic devices [3], and biomedicine [27]. Nanostructured copper sulfides can adopt a variety of compositions with two stable phases at room temperature: chalcocite (Cu_2_S) for Cu-rich phases and covellite (CuS) for Cu-deficient phases. Intermediate compositions are present between Cu_2_S and CuS, namely anilite (Cu_1.75_S), digenite (Cu_1.8_S), and djurleite (Cu_1.93_S–Cu_1.96_S). The Cu deficiency and nanostructure morphology can modulate the band gap values in the 1.0–2.6 eV range [30,31,32] and subsequently enable tuning of the optical properties. The nanostructured copper sulfides with tunable optical properties are typically synthesized with toxic chemicals using a vacuum process or a high-cost and complex process, inhibiting the development of simple synthesis methods for modulating the composition, morphology, and band gap.

This paper reports a facile solution process that can synthesize nanostructured copper sulfides at room temperature with controlled morphology and composition. Through the sulfurization process with Na_2_S, nanostructured copper sulfides were synthesized in the form of nanospheres, nanoplates, and nanoplate-based complexes with various oxidation states. The oxidation state changes exhibited transitions in the composition and morphology with changes in the Na_2_S solution concentration and reaction time, leading to tunable optical properties. Our paper proposes a novel approach for synthesizing nanostructured copper sulfides that can control their morphology, composition, and optical properties at room temperature under non-vacuum conditions.

## 2. Materials and Methods

### 2.1. Chemicals and Materials

Copper foil (99.9%, 100 mm × 300 mm, 0.1 mm thick) was purchased from Nilaco Corporation (Tokyo, Japan). Sodium sulfide (Na_2_S, ≥99%, anhydrous) was purchased from Alfa-Aesar (Ward Hill, MA, USA). Hydrochloric acid (HCl, 37%) was purchased from Carlo Erba reagents (Milano, Italy). Methanol (CH_3_OH, 99.9%) was purchased from JT-Baker (Phillipsburg, NJ, USA). Ethanol (C_2_H_5_OH, 99.9%) was purchased from Samchun Chemicals (Seoul, Korea). Acetone (C_3_H_6_O, 99.5%, extra pure) and n-hexane (CH_3_(CH_2_)_4_CH_3_, 95%, extra pure) were purchased from Daejung Chemicals & Metals (Siheung, Korea). All chemicals were used without further purification. 

### 2.2. Sulfurization of Cu Foils

To remove the thin oxide layer on the Cu foil surface, the Cu foil (99.9%, dimensions of 1 cm × 5 cm) was ultrasonicated in diluted 1% HCl solution obtained from the 37% HCl solution for 30 min and immediately washed with the acetone and hexane via ultrasonication for 10 min. The Na_2_S powder was dissolved in methanol (10 mL) and deionized water (10 mL) (1:1 *v/v*) at different concentrations (10, 5, 2.5, 1, and 0.5 mM). 

A Cu tweezer was fixed to the cap of a 30 mL vial, and the pre-treated Cu foil was firmly affixed so that the movement of the Cu foil would not affect the sulfurization reaction. For the sulfurization reaction, the well-dispersed 20 mL Na_2_S solution was rapidly injected into the vial and constantly stirred at 800 rpm. The Cu foil reacted with the Na_2_S solution with different reaction times (10 and 60 min). Subsequently, the as-reacted Cu foil was washed with ethanol at least twice to clean any residue left on its surface and dried under N_2_ atmosphere.

### 2.3. Materials Characterization

Field-emission scanning electron microscopy (FESEM) was performed using a Carl Zeiss SIGMA microscope (Carl Zeiss, Jena, Germany). The X-ray diffraction (XRD) patterns were analyzed using an AXS New D8 advance diffractometer (Bruker, Billerica, MA, USA) with a Cu-Kα radiation source and a LynxEye line detector. XRD samples were prepared by attaching the Cu foils onto zero-background quartz. Field-emission transmission electron microscopy (FETEM) was performed using a JEM-F200 microscope (Jeol, Tokyo, Japan) operating at 200 kV to identify the crystalline structures and lattice spacings of the samples. Fast Fourier transform (FFT) patterns of high-resolution transmission electron microscopy (HR-TEM) images were acquired using DigitalMicrograph software from Gatan Inc (Gatan Inc, GMS version 3.0 software, Pleasanton, CA, USA). Furthermore, X-ray photoelectron spectroscopy (XPS) was performed on a K-alpha^+^ system (Thermo Fisher Scientific, Waltham, MA, USA) with an Al K-alpha monochromatic X-ray beam. For the XPS analysis, all the samples were cut to a size of 10 mm × 10 mm. Ultraviolet-visible (UV-vis) spectra were measured using a JASCO V-700 spectrophotometer (Jasco Corp, Tokyo, Japan) from 300 to 1500 nm using an integrative sphere. To calculate the size of the particles in the FESEM images, the average values of the particle size were calculated on the nanometer scale, considering the resolution of the FESEM. 

## 3. Results

Sulfurization of Cu films was performed at room temperature through a simple solution process. Ten samples were prepared by changing the Na_2_S solution concentration from 0.5 to 10 mM and by controlling the reaction time for 10 and 60 min. These 10 samples were termed as C–M, where C refers to the Na_2_S solution concentration in mM and M refers to the reaction time in min.

**Figure 1:**
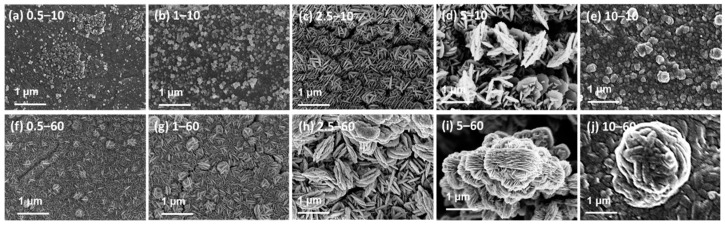
SEM images of the Cu films sulfurized using Na_2_S solution with varying concentrations (c = 0.5–10 mM) for different reaction times of 10 and 60 min; (**a**) 0.5–10, (**b**) 1–10, (**c**) 2.5–10, (**d**) 5–10, (**e**) 10–10, (**f**) 0.5–60, (**g**) 1–60, (**h**) 2.5–60, (**i**) 5–60, and (**j**) 10–60 sample.

Figure 1 shows the FESEM images detailing the morphological transition of the samples depending on the Na_2_S solution concentration and reaction time. Exposure of the Cu films in the Na_2_S solution caused the formation of nanoparticles with either nanosphere or nanoplate morphologies through the active sulfurization reaction, which did not occur for the bare Cu foil (Figure S1). With increasing reaction time, the nanoparticles increased in size and grew into nanoplates for all Na_2_S solution concentrations. For the short reaction time (10 min), the copper sulfide nanoparticles exhibited a nanosphere morphology at low solution concentrations, whereas they exhibited a nanoplate morphology at high solution concentrations. The copper sulfide nanospheres had diameters of approximately 88 nm (0.5–10 sample, Figure 1a) and 116 nm (1–10 sample, Figure 1b). The 2.5–10 (Figure 1c) and 5–10 (Figure 1d) samples exhibited a nanoplate morphology, whereas the 10–10 sample (Figure 1e) exhibited a spherical morphology.

When the reaction time was 60 min, all the samples exhibited nanoplate morphologies, and these nanoplates agglomerated into spherical shapes, forming nanoplate-based complexes. The size of the complexes was affected by the Na_2_S solution concentration, yielding large nanoplates-based complexes at high concentrations. The 0.5–60 (Figure 1f) and 1–60 (Figure 1g) samples showed that the nanoplates agglomerated to form nanoplates-based complexes with sizes of 0.198 and 0.232 μm, respectively. For concentrations higher than 2.5 mM, the spheres gradually increased (2.5–60 sample, Figure 1h). The 2.5–60 (Figure 1h), 5–60 (Figure 1i), and 10–60 (Figure 1j) samples formed large nanoplate-based complexes of approximately 1.53, 1.89, and 3.55 μm in size, respectively. Previous studies [33] on the growth mechanism of copper sulfide nanoplates reported that the initial nanoplates increased in size over long reaction times and resulted in layer-by-layer growth, forming nanoplate-based complexes, which were due to the diffusion of the sulfur ions in the sodium sulfide solution. The formation of nanoplates in the early stages and subsequent changes in their morphologies are common in the growth of copper sulfides. When Cu films were sulfurized for a long duration of 180 min (10–180 sample, Figure S2), the resultant sample comprised areas where nanospheres and nanoplates coexisted (Figure S2b). The simultaneous presence of nanospheres and nanoplates might imply that the newly exposed Cu films were sulfurized again, as the Cu films overreacted with the highly concentrated Na_2_S solution and the formed copper sulfide nanoparticles were partially peeled off.

**Figure 2:**
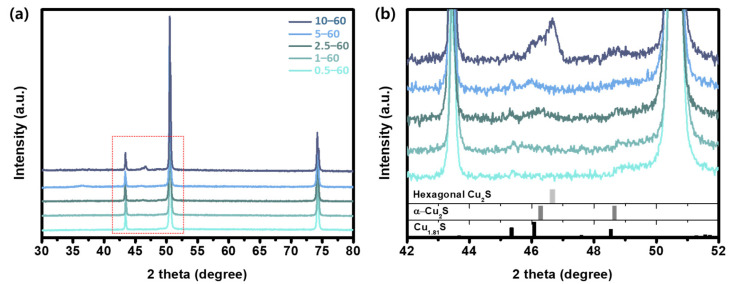
(**a**) XRD patterns of Cu films sulfurized with the reaction time of 60 min with varying Na_2_S solution concentrations (c = 0.5–10 mM) and (**b**) corresponding enlarged XRD patterns of 0.5–60, 1–60, 2.5–60, 5–60, and 10–60 samples; the bars underneath correspond to the reference data for the hexagonal Cu_2_S (PDF 01-073-6087), α-Cu_2_S (PDF 00-002-1286), and Cu_1.81_S (PDF 00-041-0959).

In addition to the morphology changes that depended on the solution concentration and reaction time, XRD patterns for the samples reacted for 60 min were analyzed to identify the crystalline structure of the copper sulfide films. The major XRD peaks of the samples ranged from 44° to 50° (Figure 2b), which were due to the main characteristic copper sulfide phases: Cu_1.81_S, α-Cu_2_S, and hexagonal Cu_2_S. Depending on the Na_2_S solution concentrations, the samples yielded different XRD results, showing that new XRD peaks were produced and that the peak intensities increased with the solution concentration. All the samples except for the 10–60 sample showed the Cu_1.81_S phase, and the samples that reacted in the solution with high concentrations of Na_2_S possessed α-Cu_2_S, hexagonal Cu_2_S, and Cu_1.81_S, whereas only hexagonal Cu_2_S was present in the 10–60 sample.

The major crystal structures of the samples detected via XRD analysis (Figure 2b) were hexagonal Cu_2_S chalcocite (PDF 01-073-6087), orthorhombic α-Cu_2_S chalcocite (PDF 00-002-1286), and tetragonal Cu_1.81_S (PDF 00-041-0959). At low Na_2_S solution concentrations in the XRD pattern of the 0.5–60 sample, distinguishing significant copper sulfide peaks from the background was difficult despite the long reaction time, which might be due to the insufficient amount captured by XRD and the sample’s amorphous structures. The XRD pattern of the 1–60 sample appeared to be the crystal structure of tetragonal Cu_1.81_S, with the diffraction peak at 45.4° corresponding to the (1 3 5) plane of the Cu_1.81_S. The XRD pattern of the 2.5–60 sample comprised peaks at 45.4° and 46.3° that were assigned to Cu_1.81_S and α-Cu_2_S, respectively. The 5–60 sample exhibited two peaks at 45.4° and 46.1°, which well-matched the (1 3 5) and (0 1 9) planes of the tetragonal Cu_1.81_S, respectively.

For the 10 mM Na_2_S solution concentration, the XRD patterns exhibited three copper sulfide phases: hexagonal Cu_2_S, α-Cu_2_S, and Cu_1.81_S. The existence of different copper sulfide phases suggests that the sample comprised mixed phases of copper sulfide, especially Cu-deficient phases, where Cu atoms do not occupy the highly symmetric positions but instead occupy a variety of lower symmetries [34]. These XRD results demonstrate that our solution process with Na_2_S solution is an efficient sulfurization method for producing Cu-deficient nanostructures using bare Cu foil at room temperature.

**Figure 3:**
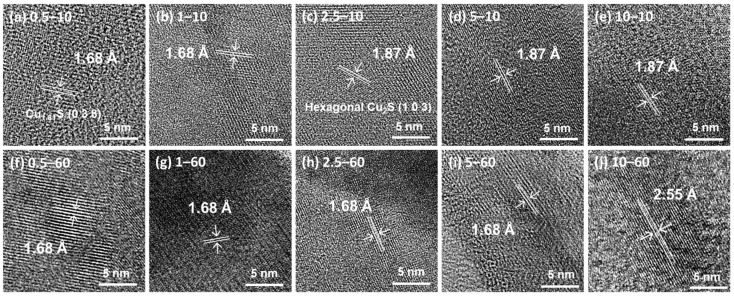
HRTEM images of the samples with varying Na_2_S concentrations (c = 0.5–10 mM) for the reaction times of 10 and 60 min; (**a**) 0.5–10, (**b**) 1–10, (**c**) 2.5–10, (**d**) 5–10, (**e**) 10–10, (**f**) 0.5–60, (**g**) 1–60, (**h**) 2.5–60, (**i**) 5–60, and (**j**) 10–60 sample.

To further investigate the crystallographic features correlated with the XRD results, HRTEM images were obtained (Figure 3). The figure shows that all the samples had clear lattice fringes that were identified as copper sulfide phases. Based on the lattice spacings of all the samples obtained from the HRTEM analyses, Cu_1.81_S, hexagonal Cu_2_S, and α-Cu_2_S were observed in the HRTEM images, as expected from the XRD results. Moreover, the HRTEM images exhibited lattice spacings of 1.68, 1.87, and 2.55 Å, which were indexed to the (0 3 8) plane of Cu_1.81_S, the (1 0 3) plane of the hexagonal Cu_2_S, and the (2 9 3) plane of α-Cu_2_S, respectively. The presence of the lattices suggests that even the samples that did not show any apparent copper sulfide phase peaks possessed crystal structures. All the samples exhibited weak diffraction peaks in XRD analyses mainly due to the small amount of nanoparticles formed, independent of the crystallinity.

**Figure 4:**
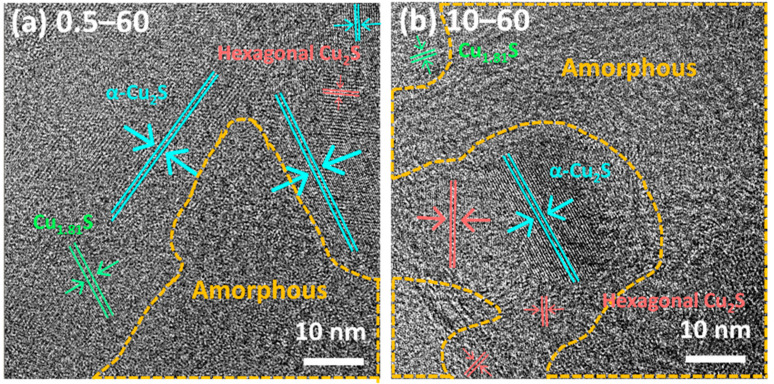
HRTEM images of the samples with c = 0.5 and 10 mM of Na_2_S solution for the reaction time of t = 60 min: (**a**) 0.5–60 sample and (**b**) 10–60 sample. The yellow dashed regions indicate the amorphous structures, and the red, cyan, and green lines denote the hexagonal Cu_2_S, α-Cu_2_S, and Cu_1.81_S phases, respectively.

The crystalline grains can be identified from the HRTEM images in Figure 4 and the FFT patterns of the HRTEM images in Figure S3, which specify the relative orientations of each grain with respect to the others. The grain orientations in all the samples were identified using HRTEM images and their FFT patterns, and distinctive colors (green, red, and cyan) were used to represent the three different copper sulfide phases (Cu_1.81_S, hexagonal Cu_2_S, and α-Cu_2_S, respectively). All the samples exhibited the coexistence of Cu_1.81_S, hexagonal Cu_2_S, and α-Cu_2_S phases regardless of the Na_2_S solution concentration and reaction time, and two samples are shown in Figure 4 as examples. The two HRTEM images of the 0.5–60 (Figure 4a) and 10–60 (Figure 4b) samples denote that the grains had amorphous structures, represented by yellow dash lines, and crystalline structures. Both samples contained crystal structures of Cu_1.81_S, hexagonal Cu_2_S, and α-Cu_2_S. Although the 0.5–60 sample had an insufficient quantity to be detected in XRD, it exhibited all three crystal structures (Figure 4a and Figure S3a). The 10–60 sample (Figure 4b and Figure S3b) also exhibited all three crystal structures consistent with the XRD results, which suggest that various crystalline phases are present at high solution concentrations. The reason for the extremely weak peaks in the XRD analyses is the partial amorphous structure of the samples, as confirmed by the HRTEM results in Figure 4a. The 10–60 sample (Figure 4b) exhibited amorphous structures despite the intense peaks in the XRD patterns.

**Figure 5:**
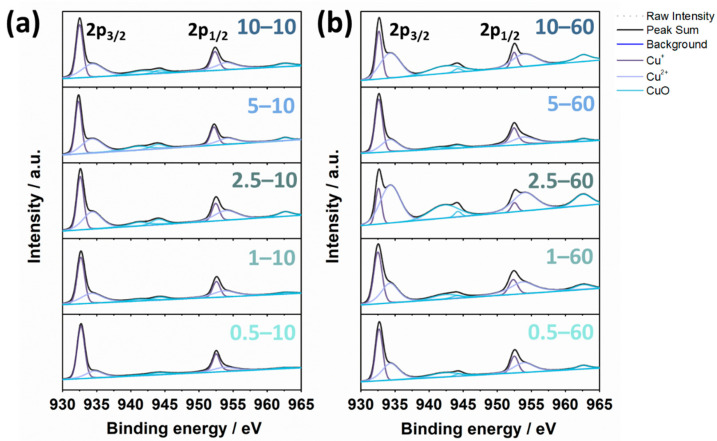
XPS spectra of the samples sulfurized from the Na_2_S solution with different concentrations (c = 0.5–10 mM) for reaction times of 10 and 60 min: the Cu 2*p* region of the samples reacted for (**a**) 10 min and (**b**) 60 min.

While the XRD and HRTEM results indicated the crystalline Cu_2_S and Cu_1.81_S phases, the XPS analysis of the film surfaces also suggested the presence of Cu_2_S or CuS phases (Figure 5). The Cu 2*p* XPS spectrum exhibited two peaks at 932.7 and 952.6 eV that could be Cu 2*p*_3/2_ and 2*p*_1/2_, respectively [35,36]. The binding energy difference between the two peaks was approximately 19.9 eV, which is in good agreement with the values reported in the literature [37]. The Cu 2*p*_3/2_ spectra of all the samples were deconvoluted into two characteristic peaks, with signals at 932.6 and 934.4 eV, which are attributed to Cu^+^ and Cu^2+^, respectively [35,38]. The binding energies of Cu^+^ and Cu^2+^ barely changed for all the samples, suggesting that the chemical bonds between the copper and sulfur atoms were retained. The existence of the Cu^2+^ ions may indicate that they were formed in the solution during the sulfurization process and slightly oxidized after the reaction. Changes in the fitted areas did not vary with the solution concentrations, but they were affected by the reaction times. The area ratios of Cu^+^/Cu^2+^ decreased with increasing reaction time, indicating that the reaction time affects the amount of Cu^+^ and Cu^2+^ species present on the surface. In terms of the peak binding energies and the fitted areas of the deconvoluted peaks, the calculated oxidation states for the samples reacted for 10 min were compared to those for the samples reacted for 60 min. For all solution concentrations, the oxidation states of the samples reacted for 60 min were higher than those of the samples reacted for 10 min (Figure S5). Through an accurate peak-fitting on the Cu 2*p* spectra, it was confirmed that the 2*p*_3/2_ areas of Cu^+^ and Cu^2+^ were not twice as large as 2*p*_1/2_ areas of Cu^+^ and Cu^2+^, respectively. The results of comparing the areas of 2*p*_3/2_ and 2*p*_1/2_ indicate that the peaks of copper metal, asymmetric Cu^+^, and copper hydroxide can also exist at 932.6–932.8 [39,40], 933.14 [41], and 935.65 eV [42,43], respectively, which might be due to the exposure of the films to air. The deconvoluted XPS spectra comprised a series of satellite peaks with binding energies at 942.4, 944.4, and 962.8 eV, corresponding to the presence of CuO (Figure 5) [44,45]. The satellite peaks of all the samples revealed a small fraction of CuO, and the area of the satellite peaks varied depending on the reaction time. The fitted area of the satellite peaks for the 60 min samples was larger than that for the 10 min samples, indicating that the surfaces of the samples were oxidized upon exposure to air during the long reaction time. Overall, the oxidation states of all the samples increased over the long reaction time (60 min) with the changing Cu^+^/Cu^2+^ area ratio over time, maintaining the chemical bond between the Cu and S atoms. These XPS results imply that the oxidation state can be modulated on the copper sulfide film surface by controlling the reaction time. For the presence of S 2*p*, the XPS results in Figure S5 show that the peak at ~162.1 eV can be ascribed to S^2−^ 2*p*_3/2_ and the peak at ~163.2 eV to S^2−^ 2*p*_1/2_ [46,47,48,49]. The peaks at 166.7~168.7 eV were associated with oxidized sulfur species such as sulfonate and sulfate groups [50,51]. For all the samples, the binding energies of S^2−^ peaks were maintained, while the oxidized sulfur species varied. The co-existence of the S^2−^ 2*p*, Cu^2+^ and Cu^+^ 2*p* peaks demonstrated that the copper sulfide with Cu-deficient phases was controlled by the reaction time.

**Figure 6:**
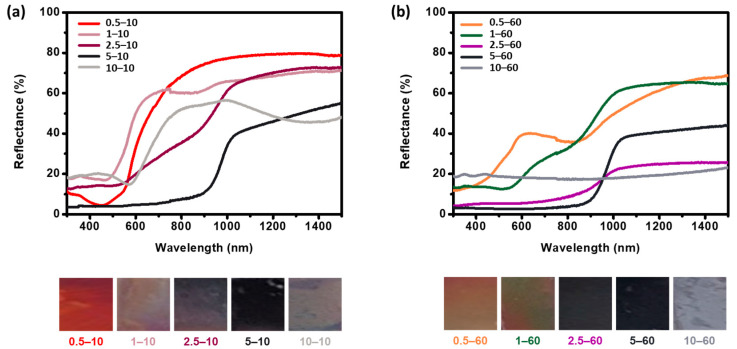
UV-vis spectra and photographs of the samples sulfurized in various Na_2_S solution concentrations for (**a**) 10 min and (**b**) 60 min.

The optical properties of the copper sulfide films were investigated via optical reflectance measurements (Figure 6). When two variables in the experiment were controlled (Na_2_S solution concentrations and reaction times), each film exhibited unique colors that were reflected in the UV-vis reflectance spectra. For the reaction time of 10 min (Figure 6a), the samples were found to be highly reflective in the near infrared (NIR) region, while the samples reacted for 60 min (Figure 6b) were less reflective at all solution concentrations. This reduction in reflectivity from 10 to 60 min is related to the localized surface plasmon resonances [52,53], which stem from the free-carrier density [43]. The optical band gaps of copper sulfide can be modulated by changing the compositions, and they reportedly exhibit band gap values of 1.0 to 2.6 eV with decreasing Cu_x_S composition from x = 2 to 1.8 [53,54,55]. Based on the increase in the band gap with the decreasing x value of Cu_x_S, the differences in the optical band gaps affected the optical absorption, showing that Cu-deficient compositions exhibited stronger absorption in the NIR region than the Cu-rich compositions. The XPS analyses showed that the oxidation states of the samples increased with the reaction times. The reduced reflectivity values of the samples for the longer reaction times are consistent with the higher oxidation states obtained from the XPS analyses (Figure 6). Moreover, the decrease in reflectivity with increasing reaction times might be attributed to the enhanced scattering with increasing crystallite sizes of the copper sulfide nanoparticles [43]. Through the inverse relationship between crystallite sizes and band gap values, the lower reflectivity values of the 60 min samples might possess low band gaps due to their small grain sizes, as seen in the FESEM images. Regardless of the reaction times, at high concentrations, the samples with dark colors showed low reflectivity in the visible region, whereas at low concentrations, the samples with bright colors, such as red, yellow, and yellowish-green, exhibited high reflectivity. The differences in reflectivity were shown as the differences of exterior colors, indicating that the samples with the low reflectivity values exhibited colors close to black [56].

Consequently, when the concentrations were controlled, the reactions in high concentration solutions indicated a decrease in reflectivity, which might be due to the increased crystallite sizes resulting in enhanced scattering. When the reaction time was controlled, the long reaction time (t = 60 min) resulted in Cu-deficient phases of copper sulfide, subsequently decreasing the reflectivity. The optical properties as well as the sizes, morphologies, compositions, and phases of the nanostructured copper sulfide can be controlled through this sulfurization process. In addition, the sulfurization process can be expected to enhance the electrical and mechanical properties [30,57,58], which may provide the opportunity for nanostructured copper sulfide films to be used as photocatalysts for energy conversion.

## 4. Conclusions

This paper presents a facile synthesis method for preparing nanostructured copper sulfide via the sulfurization of Cu films at room temperature by controlling the Na_2_S solution concentration and reaction time. Unlike other synthesis methods that require high temperatures and long reaction times, the proposed solution process with Na_2_S enables reactive sulfurization at room temperature with a short reaction time (<60 min). The Na_2_S solution concentration and reaction time were demonstrated as the key factors that determine the morphology and composition of the nanostructured copper sulfide, confirming that the oxidation states were also modulated. Moreover, by comparing the optical properties of the copper sulfide films with Cu-deficient and Cu-rich phases, we determined that the increased oxidation states play a significant role in the reduction in reflectivity in the NIR region. Our simple sulfurization process with Na_2_S provides a new pathway for further improving the nanostructured copper sulfide synthesis process by controlling the morphology, composition, and optical properties without requiring high temperatures and long reaction times.

## Data Availability

The data presented in this study are available on request from the corresponding author.

## Supplementary Materials

The following are available online at https://www.mdpi.com/article/10.3390/nano11092317/s1. Figure S1. A bare Cu foil without the sulfurization process. Figure S2. SEM images of the Cu film sulfurized in 10 mM Na_2_S solution for 180 min (10–180 sample). The scale bar is 1 μm. Figure S3. Fast Fourier Transform (FFT) patterns of the high-resolution images showing the hexagonal Cu_2_S, α-Cu_2_S, and Cu_1.81_S phases (a) in the 0.5–60 and (b) 10–60 sample in Figure 4. Figure S4. The oxidation states of the Cu films sulfurized for the reaction times of t = 10 and 60 min in the various Na_2_S solution concentrations (c = 0.5, 1, 2.5, 5, and 10 mM). Figure S5. XPS spectra of the samples sulfurized from the Na_2_S solution with different concentrations (c = 0.5–10 mM) for reaction times of t = 10 and 60 min: the S 2*p* region of the samples reacted for (a) 10 min and (b) 60 min.
