# Supplementary material for: Facile Sulfurization under Ambient Condition with Na2S to Fabricate Nanostructured Copper Sulfide
Source: Nanomaterials (Basel). 2021 Sep 6;11(9):2317. doi: 10.3390/nano11092317 (PMC8471496; doi:10.3390/nano11092317)
Supplement: Supplementary file 1 [file nanomaterials-11-02317-s001.zip › nanomaterials-1351287-supplementary.pdf]

Supporting Information

Facile Sulfurization under Ambient Condition with Na_2S to Fabricate Nanostructured Copper Sulfide

Eunseo Hwang, Yoonsu Park, Jongbae Kim, Taejong Paik, and Don-Hyung Ha*

School of Integrative Engineering, Chung-Ang University, Seoul 06974, Republic of Korea
nseo4236@cau.ac.kr (E.H.); ung2150@cau.ac.kr (Y.P.); jong3070@cau.ac.kr (J.K.); paiktae@cau.ac.kr (T.P.)
* Correspondence: dhha@cau.ac.kr

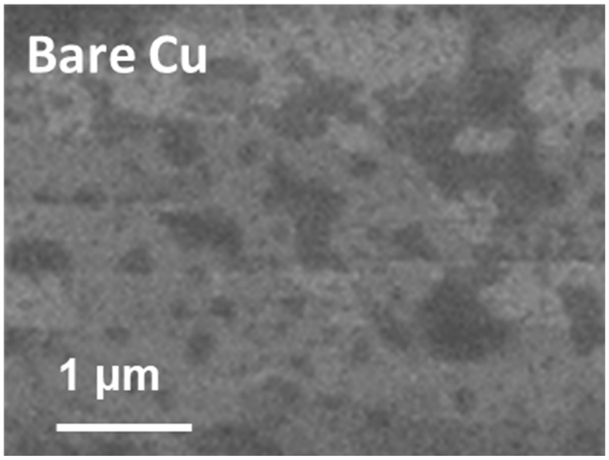

Figure S1. A bare Cu foil without the sulfurization process.

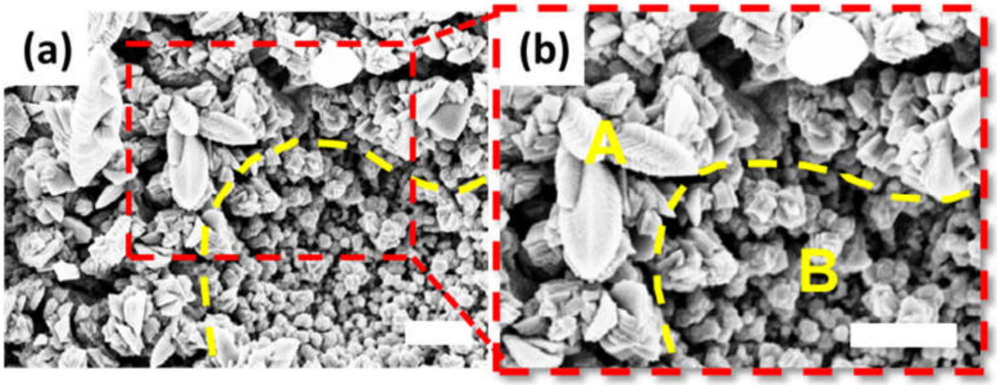

Figure S2. SEM images of the Cu film sulfurized in 10 mM Na_2S solution for 180 min (10–180 sample). The scale bar is 1 μm.

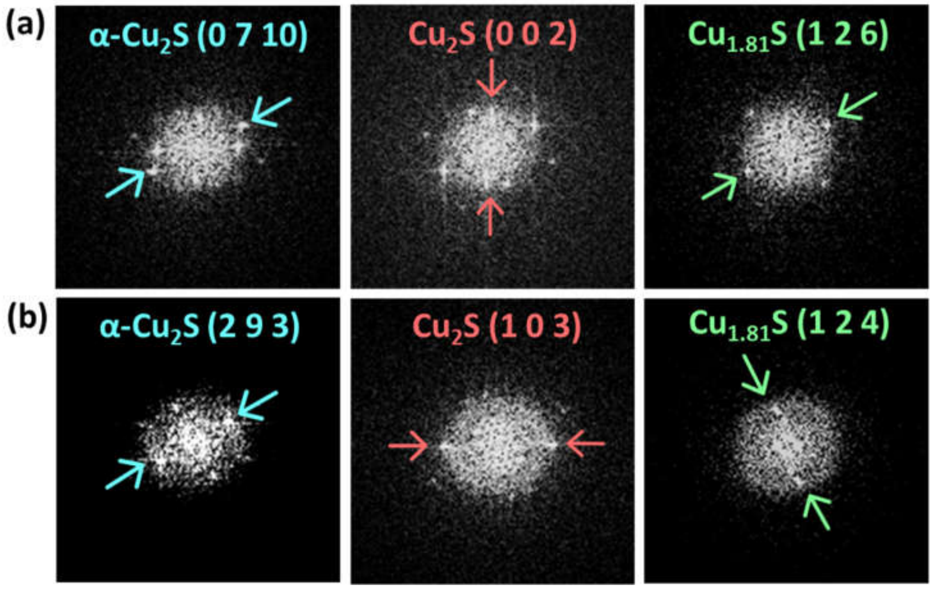

Figure S3. Fast Fourier Transform (FFT) patterns of the high-resolution images showing the hexagonal Cu_2S, α-Cu_2S, and $Cu_{1.81}S$ phases (a) in the 0.5–60 and (b) 10–60 sample in Figure 4.

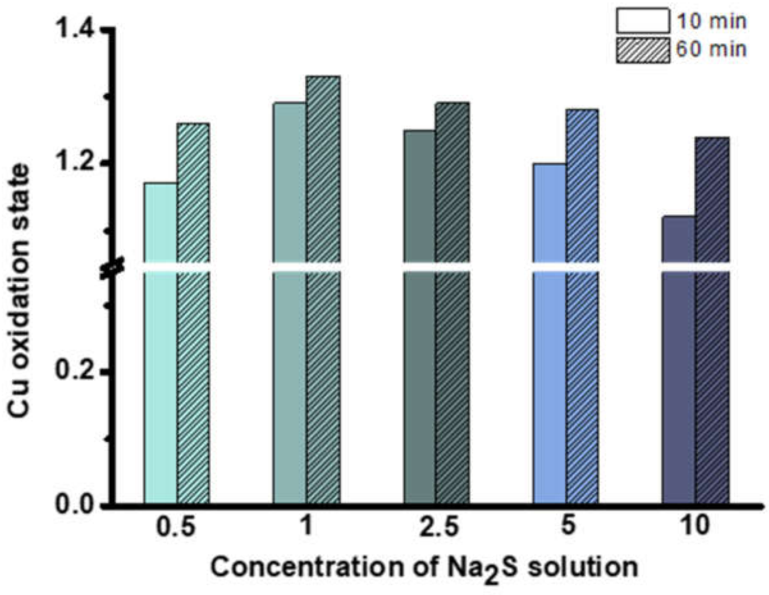

Figure S4. The oxidation states of the Cu films sulfurized for the reaction times of t = 10 and 60 min in the various Na_2S solution concentrations (c = 0.5, 1, 2.5, 5, and 10 mM).

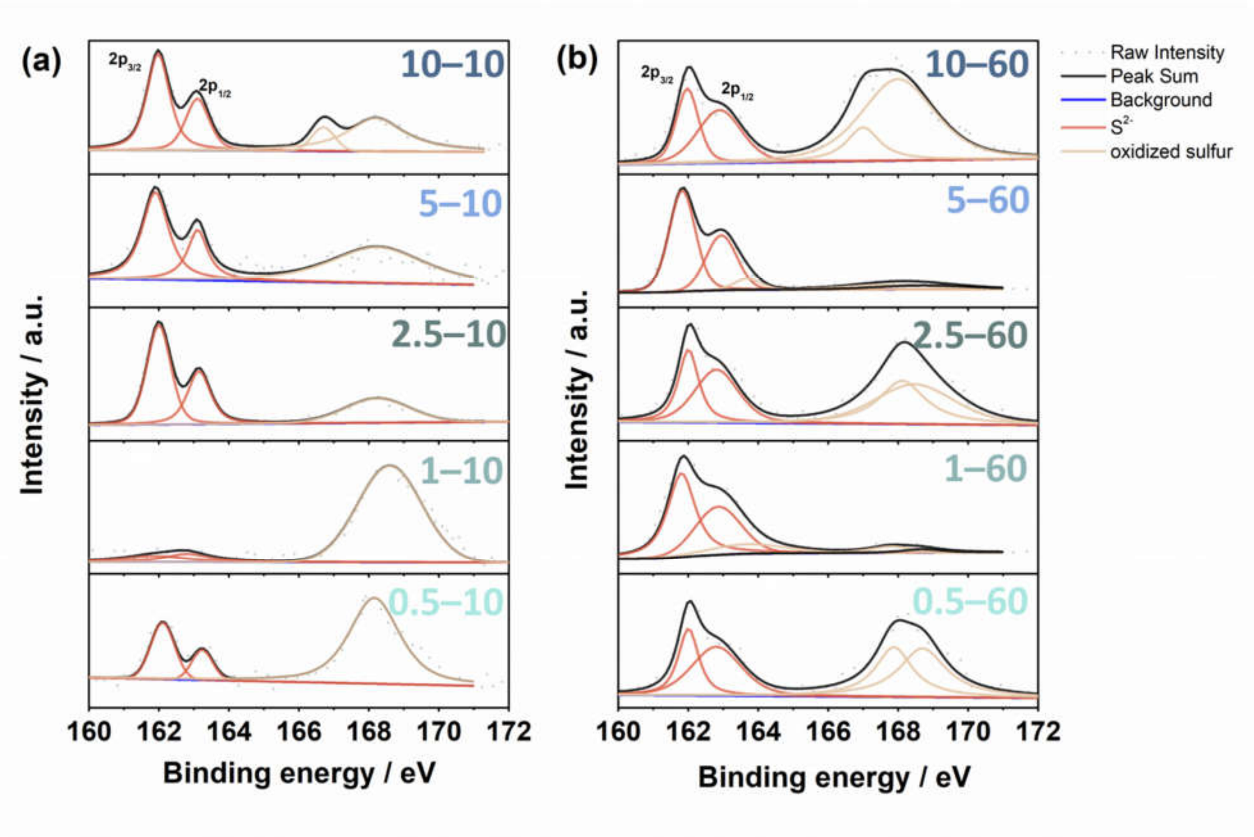

Figure S5. XPS spectra of the samples sulfurized from the Na_2S solution with different concentrations (c = 0.5–10 mM) for reaction times of t = 10 and 60 min: the S 2*p* region of the samples reacted for (a) 10 min and (b) 60 min.
